# MAT1A activation of glycolysis to promote NSCLC progression depends on stabilizing CCND1

**DOI:** 10.1038/s41419-024-07113-7

**Published:** 2024-10-22

**Authors:** Shengping Shen, Ruili Liu, Jiazheng Huang, Yingjia Sun, Qiang Tan, Qingquan Luo, Ruijun Liu

**Affiliations:** 1 Shanghai Lung Tumor Clinical Medicine Center, Shanghai Chest Hospital, Shanghai Jiao Tong University, Shanghai, 200030 PR China; 2 Department of Stomatology, Ordos Central Hospital, Ordos, 017000 Inner Mongolia PR China

**Keywords:** Non-small-cell lung cancer, Ubiquitylation

## Abstract

Non-small cell lung cancer (NSCLC) remains a cause for concern as the leading cause of cancer-related death worldwide. Amidst ongoing debates on the role and mechanisms of methionine adenosyltransferase 1A (MAT1A) in cancer, our study sheds light on its significance in NSCLC. Leveraging TCGA database and immunohistochemical staining, we systematically analyzed MAT1A expression in NSCLC, uncovering its marked upregulation. To unravel the functional and mechanistic underpinnings, we implemented stable knockdown of MAT1A in NSCLC cell lines. Our findings converged to demonstrate that suppression of MAT1A expression effectively impeded the proliferation and migratory capabilities of NSCLC cells, while concurrently enhancing apoptosis. Mechanistically, we discovered that MAT1A depletion accelerated the degradation of CCND1, a key cell cycle regulator, through S-phase kinase-associated protein 2 (SKP2)-mediated ubiquitination. Notably, CCND1 emerged as a crucial MAT1A partner, jointly orchestrating glycolytic metabolism in NSCLC cells. This intricate interplay suggests that MAT1A promotes NSCLC progression by safeguarding CCND1 protein stability and activating glycolytic pathways, thereby sustaining tumorigenesis. In summary, our study not only identifies MAT1A as a prognostic marker for poor survival in NSCLC patients but also elucidates its mechanistic contributions to cancer progression. These findings pave the way for the development of targeted therapies aimed at disrupting the deleterious MAT1A-CCND1-glycolysis axis in NSCLC.

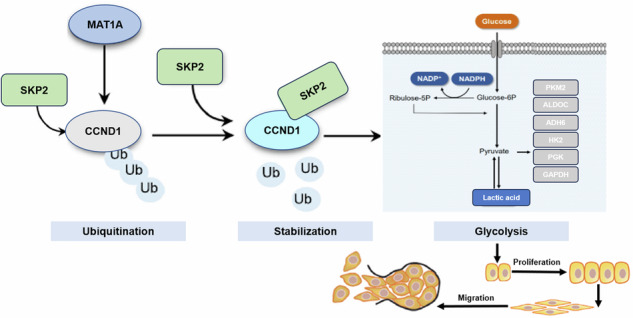

## Introduction

Lung cancer, a highly prevalent and fatal malignancy, poses a significant threat to global human health [1]. Among its various forms, non-small cell lung cancer (NSCLC) comprises approximately 80% of all lung cancer cases, making it the most prevalent subtype. This category encompasses squamous cell carcinoma, adenocarcinoma, and large cell carcinoma [2]. A major challenge lies in the fact that the majority of NSCLC patients are diagnosed at intermediate to advanced stages, resulting in a dismal five-year survival rate [3]. Traditionally, surgical intervention, coupled with preoperative or postoperative adjuvant chemotherapy and radiotherapy, has been the cornerstone of treatment strategies aimed at enhancing cure rates and prolonging survival for NSCLC patients [4]. However, despite these efforts, the occurrence of recurrence, metastasis, and undesirable toxic side effects underscores the limitations of these conventional approaches in fully meeting patient needs [5]. Notably, with Next-Generation Sequencing (NGS) becoming a standard tool for targeted therapies in well-recognized subtypes of NSCLC. For example, Kästner et al., provide comprehensive, high-quality multi-molecular diagnostics and standardized personalized treatment recommendations for patients with advanced NSCLC [6]. Hendricks et al. provided specific clinical diagnosis, treatment, and follow-up for oncogene-dependent metastatic NSCLC [7]. Despite significant advancements in the treatment of NSCLC and improved patient outcomes, the high cost remains a barrier for most patients to access these benefits. Therefore, there is an urgent need to continually explore new molecular targets in order to facilitate widespread utilization of targeted therapies against NSCLC.

Methionine adenosyltransferases (MATs), essential enzymes in the biosynthesis of S-adenosylmethionine (SAM), occupy a pivotal position within the methylation cycle [8]. In mammals, MAT isozymes are composed of distinct catalytic subunits, MAT1A and MAT2A, which encode for MATα1 and MATα2, respectively, each with distinct physiological and pathological implications [9]. Recent research has shown a spotlight on MAT1A, particularly its involvement in the metabolism and progression of various cancer types. MAT1A functions as a tumor suppressor in cholangiocarcinoma, where its encoded protein acts as a co-repressor of E-box elements, inhibiting the expression of oncogenic genes like c-MYC, MAFG, and c-MAF [10]. Furthermore, reduced MAT1A expression has been correlated with poor prognosis in hepatocellular carcinoma (HCC), suggesting its potential as a prognostic biomarker [11]. Notably, the concurrent downregulation of MAT1A and upregulation of MAT2A represents a crucial prerequisite for HCC metastasis [12, 13]. A recent study suggested that MAT1A as a tumor suppressor in HCC could inhibit NF-κB activity [14]. Given the emerging significance of MAT1A in cancer biology, our research delves into the unexplored realm of its potential role and mechanism in NSCLC, a malignancy with high morbidity and mortality. Of particular interest is the interplay between MAT1A and the methylthioadenosine (MTA) pathway, disrupted in MTAP-deleted cancers. MTAP loss leads to MTA accumulation, a metabolic alteration that has been targeted in novel therapeutic strategies for MTAP-deficient tumors [15, 16]. Therefore, elucidating the relationship between them in NSCLC may uncover novel insights into disease progression and therapeutic vulnerabilities.

In this context, we aim to investigate the functional significance of MAT1A in NSCLC and the potential of MAT1A as a novel therapeutic target or biomarker. By integrating our findings with existing knowledge on NSCLC, we hope to contribute to the development of more effective and precise treatment strategies for NSCLC.

## Materials and methods

### Tissue sample collection and immunohistochemical (IHC) staining

A lung cancer tissue microarray containing 77 lung cancer tumor tissues and 86 para-carcinoma tissues were obtained from Shanghai Yibeirui Bioscienceres Co., Ltd (Cat. YBR-HLug104-M002). The included samples included tissue from patients diagnosed with NSCLC, while those treated with radiation and chemotherapy or with other underlying conditions were excluded. Approval for this study was provided by the Ethics committee of Shanghai Chest Hospital (Approval No. KS23041). Informed consent was obtained from each lung cancer patient. Briefly, slides were deparaffinized in xylene and rehydrated in ethanol with different concentrations. Antigen retrieval was performed by heating sections for 10 min with citrate buffer (pH 6.0) followed by eliminating endogenous peroxidase using 3% H_2_O_2_ for 5 min at room temperature. Subsequently, slides were incubated with normal serum for 20 min to avoid non-specific background. After washing with PBS for 3 times, slides were incubated with the primary antibody (anti-MAT1A, Biorbyt, Cat. No. orb341137; anti-CCND1, Bosterbio, Cat. No. BM4272; anti-ALDOC, Proteintech Group, Cat. No. 14884-1-AP; anti-Ki-67, Baso, Cat. BA4041) overnight at 4 °C and then incubated with secondary antibody goat anti-rabbit IgG (Abcam, Cat. ab97080) for 30 min at room temperature. Finally, the slides were developed with DAB, counterstained with hematoxylin (Baso, Cat. BA4041), and observed under an optical microscope. The intensity of positive-staining cytoplasm or nucleus of target gene was scored on a scale of 0‐3 (0, negative; 1, light brown; 2, medium brown; and 3, dark brown), which was multiplied by the corresponding value of positive percentage (1, less than 25%; 2, 25%‐50%; 3, 50%‐75%; and 4, more than 75%).

### Cell lines and transfection

BEAS-2B is a normal human lung epithelial cell line, A549, NCI-H1299, NCI-H1437 and NCI-H1944 are human NSCLC cell lines [17]. These cells were all purchased from the Procell Life Science & Technology Co., Ltd (Wuhan, China) and examined by STR. All cells were cultured in 90% RPMI-1640 (Corning, USA) supplemented with 10% FBS (Invitrogen, USA) in a humidified incubator at 37°C with 5% CO_2_.

The lentiviral vectors of short hairpin RNA (shRNA) targeting MAT1A or CCND1 and MAT1A-overexpressing lentivirus were synthesized by Shanghai Yibeirui Bioscienceres (Shanghai, China). A scramble shRNA vector and empty vector were used as negative control. Briefly, the RNA interference sequences were inserted into BR-V-108 or LV-004 lentivirus vector (Shanghai Bioscienceres, Co., Ltd, China) according to the manufacturer’s instruction. The positive cloned plasmids were amplified and then transfected to 293T cells. Virus particles were harvested 48 h after transfection. Recombinant lentivirus plasmids were transfected into A549 and NCI-H1299 cell lines by using Lipofectamine 3000 according to the manufacturer’s instructions. Target sequences and shRNA sequences mentioned above were shown in Supplementary Table S1.

### Quantitative real-time PCR (qPCR)

Total RNA was isolated from A549 and NCI-H1299 cells with TRIzol® reagent (Invitrogen, USA) according to the manufacturer’s protocol. A cDNA reverse transcription kit (TransGen Biotech, China) was used to generate cDNA from mRNA. qPCR was performed using a SYBR Green kit (Vazyme, China) with the Biosystems 7500 Sequence Detection system (Applied Biosystems, USA) according to the manufacturer’s protocol. GAPDH was used as the reference gene. Gene expression was analyzed using the 2^−ΔΔCq^ method. Primer sequences are listed in Supplementary Table S2.

### Western blot (WB) analysis

A549 and NCI-H1299 cells were lysed in RIPA buffer and the protein concentration was quantified with a BCA Protein Assay kit (HyClone-Pierce, USA). Equal amounts of proteins (20 µg) were separated by 10% sodium dodecyl sulfate polyacrylamide gel electrophoresis (SDS‐PAGE) and transferred onto polyvinylidene difluoride (PVDF) membranes. The PVDF membranes were blocked with 5% skim milk for 1 h at room temperature and then incubated with primary antibodies overnight at 4 °C. After washing with TBST buffer for three times, the membranes were incubated with secondary antibodies at room temperature for 1 h. The relative protein levels were visualized by ECL plus TM WB system kit (Millipore, USA). Antibodies used for WB were listed in Supplementary Table S3.

### Co-immunoprecipitation (Co-IP) and ubiquitination assays

A549 cell were solubilized in ice-cold IP buffer and the total proteins were quantified by BCA kit (HyClone-Pierce, USA). 1.0 mg total proteins were incubated with anti-CCND1 or anti-Ubiquitin at 4 °C overnight followed by incubation with 20 μL agarose beads for 2 h at 4 °C. The immune complexes were washed using IP buffer and boiled with SDS‐PAGE loading buffer. Finally, the complexes were subjected to CCND1 ubiquitination detection by WB analysis. Antibodies used in Co-IP were listed in Supplementary Table S3.

A549 cells transfected with shMAT1A, SKP2 or negative control were treated with cycloheximide (CHX) or MG-132 at 10 μmol/L for 8 h. The CCND1 protein levels were detected at indicated time points by WB analysis.

### Cell proliferation assay

Cell proliferation was assessed by Celigo cell counting assay and cell counting kit-8 (CCK-8) assay. For the Celigo cell counting assay, A549 and NCI-H1299 cells were plated in 96‐well plates at a density of 2 ×10^3^ cells per well at 72 h after transfection. The GFP‐positive cells were counted in 5 consecutive days by Celigo image cytometer (Nexcelom Bioscience). The cell proliferation curve was then graphed based on cell counts.

For the CCK-8 assay, cells were seeded into 96-well plates at the density of 2 ×10^3^ cells per well and then cultured in a 5% CO_2_ incubator with or without the PI3K inhibitor (LY294002, 40 μM, Cat. S1105, Selleck) overnight. The following day, 10 μL/well CCK-8 reagent was added, and the cells were incubated for 4 h. Finally, the absorbance was measured at 450 nm using a microplate reader (Tecan infinite, Switzerland).

### Flow cytometry

Cell apoptosis and cell cycle progression were assessed utilizing flow cytometry. Following 72 h of transfection, A549 and NCI-H1299 cells, harvested from the logarithmic growth phase, were centrifuged, washed, and resuspended in complete culture medium. For the apoptosis assay, cells were gently rinsed with chilled D-Hank solution (pH 7.2–7.4) at 4 °C and subsequently resuspended in 200 μL of 1X binding buffer. Next, cells were stained with 10 μL of Annexin V-APC and propidium iodide (PI) for 10 minutes in the dark to allow for specific labeling of apoptotic cells.

In contrast, for the cell cycle analysis, cells were first washed with phosphate-buffered saline (PBS) and then fixed by incubation in 70% ethanol at 4 °C for 60 min. Following fixation, cells were permeabilized and stained with PI staining buffer according to the manufacturer’s protocol. This step facilitated the quantification of DNA content, which is indicative of the cell cycle stage.

Finally, both apoptosis and cell cycle distributions were quantitatively determined using a flow cytometer (Millipore, USA). The percentages of apoptotic cells, as well as the distribution of cells across different phases of the cell cycle, were analyzed and reported accordingly.

### Cell migration

Cell migration activity was assessed by wound-healing assay and Transwell assay. For the wound-healing assay, A549 and NCI-H1299 cells were plated in a 96-well plate at the density of 3 ×10^4^ cells per well and cultured with low concentration serum overnight. The following day, the lower central of the 96-well plate was aligned with the scraping device and gently pushed upward to form a scratch. Serum-free culture medium was used to rinse the cells gently, and culture medium with low concentration serum was added. The microscopic observation was performed at 0 h, 8 h, 24 h, 36 h and 72 h in the same field and photographed, respectively.

For the Transwell assay, approximately 100 μL serum-free medium containing 1.5 ×10^5^ A549 and NCI-H1299 cells were added into the upper chamber of a 24 well plate, while lower chamber was filled with 600 μL of medium containing 15% FBS. Cells were cultured in a 5% CO_2_ incubator for 18 h. Subsequently, non-migrated cells were removed and migrated cells were stained with crystal violet followed by examination under a microscope.

### Measurement of cellular glycolysis

The cellular glycolysis indicators, including glucose (Beijing Solarbio Science & Technology, Cat. No. BC2500), lactic acid (Beijing Solarbio Science & Technology, Cat. No. BC2230), pyruvate (PA; Beijing Solarbio Science & Technology, Cat. No. BC2205), 3-phosphoglycerate kinase (PGK; Beijing Solarbio Science & Technology, Cat. No. BC2255), and glyceraldehyde 3-phosphate dehydrogenase (GAPDH; Beijing Solarbio Science & Technology, Cat. No. BC2215), typically involves the use of specialized kits tailored for each analyte.

Briefly, A549 cells were lysed to release intracellular contents, and the lysates were typically clarified by centrifugation to remove debris. Aliquots of the prepared samples are mixed with the appropriate assay buffer and substrates/reagents provided in the kit, following the manufacturer’s instructions. The reaction mixtures were incubated for a specified time and temperature to allow for the enzymatic reactions to proceed. The resulting product is quantified using a spectrophotometer depending on the assay chemistry.

### Mice xenograft model

All BALB/c nude mice (4-week-old) were purchased from Beijing Vital River Laboratory Animal Technology Co., Ltd. (Approval No. SCXK 2016-0006) and housed in a specific-pathogen-free environment. The in vivo xenograft assay was approved by Ethics Committee of Shanghai Chest Hospital. The corresponding NSCLC cell model was subcutaneously injected into the dorsal region of nude mice to establish the xenograft tumor model, which included following groups: shCtrl (*n* = 6) and shMAT1A (*n* = 6); NC (*n* = 6), MAT1A (*n* = 6), shCCND1 (*n* = 6) and MAT1A+shCCND1 (*n* = 6). Tumor growth was monitored at the specified time and according to the formula (V = π/6 × L × W^2^ (W, width at the widest point; L, vertical width)) and weighing to estimate tumor size. After the mice were killed by cervical dislocation, the tumor body was taken, photographed and weighed. As previously mentioned, protein expression in tumor tissues was analyzed by HE and IHC staining and WB.

### RNA-seq analysis

To investigate the role of MAT1A in the progression of NSCLC, we extracted total RNA from shCtrl (*n* = 3) and shMAT1A (*n* = 3) NCI-H1299 cell lines, and then performed RNA sequencing (RNA-seq) according to the manufacturer’s instructions. The difference in the gene expression was considered significant when the |Fold Change|≥ 1.3 and *p* value < 0.05. The functional interpretation of differentially expressed genes (DEGs) was annotated using Ingenuity Pathway Analysis (IPA).

### Statistical analysis

Statistical analysis was performed by Graphpad Prism 8.04 software. The data were shown as the mea*n* ± standard deviation (SD). Statistical analysis between two groups was performed by two-tailed Student’s *t* test. *P* value of <0.05 was considered significantly difference.

## Results

### MAT1A is abundantly expressed in NSCLC

**Fig. 1:**
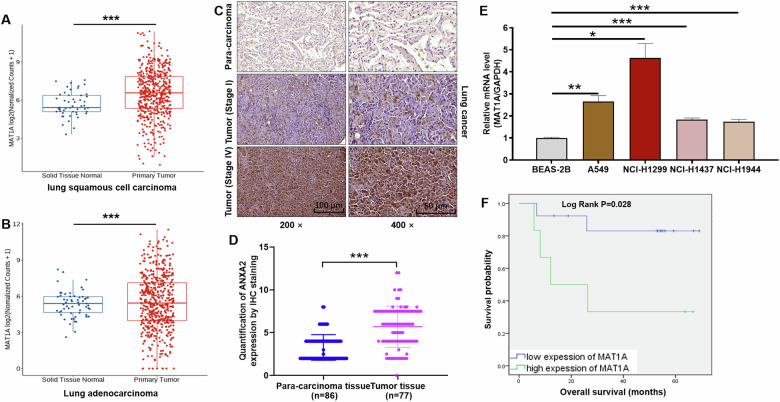
MAT1A was abundantly expressed in NSCLC and associated with prognosis of NSCLC patients. **A**, **B** Based on The Cancer Genome Atlas (TCGA) program, the expression of MAT1A in patients with lung squamous cell carcinoma and lung adenocarcinoma was analyzed. **C** Representative IHC staining images of MAT1A in human NSCLC tissues and para-carcinoma tissues. **D** IHC quantification of the expression of MAT1A in tumors and para-carcinoma tissues of NSCLC patients. **E** MAT1A expression levels in human NSCLC cell lines (A549, NCI-H1299 and NCI-H1944) and normal human lung epithelial cell line (BEAS-2B) were determined by qPCR. Results were shown as mean ± SD. **F** Correlation between MAT1A expression and overall survival of NSCLC was analyzed by Kaplan-Meier survival analysis with a log-rank test. *p < 0.05, **p < 0.01, ***p < 0.001.

Utilizing data from The Cancer Genome Atlas (TCGA) program, we conducted a comprehensive analysis of MAT1A expression in patients with lung squamous cell carcinoma (LUSC) and lung adenocarcinoma (LUAD), as depicted in Fig. 1A, B. Our findings revealed a statistically significant upregulation of MAT1A expression in both LUSC and LUAD tissues compared to normal lung tissues (*p* < 0.001), underscoring its potential role in lung carcinogenesis.

To validate these bioinformatic observations, immunohistochemical (IHC) staining was performed on tissue microarrays comprising tumor (*n* = 77) and adjacent non-tumor (para-carcinoma, *n* = 86) tissues from patients with NSCLC. The IHC images clearly demonstrated a marked contrast, with weak positive signals in para-carcinoma tissues contrasting sharply with robust MAT1A staining in NSCLC tissues. Furthermore, we noted a trend towards increased MAT1A expression in advanced tumor stages (stage IV) compared to early-stage tumors (stage I), as illustrated in Fig. 1C. Statistical analysis corroborated this observation, highlighting a significant difference in MAT1A overexpression between tumor tissues (56.5%) and normal tissues (11.6%) (*p* < 0.001), as presented in Fig. 1D and Table 1.

**Table 1:** Expression patterns of MAT1A in lung cancer tissues and para-carcinoma tissues were revealed by immunohistochemistry analysis.

MAT1A expression	Tumor tissue		Para-carcinoma tissue		*P* value
	patients	Percentage	patients	Percentage	
Low	34	44.2%	76	88.4%	*P* < 0.001
High	43	55.8%	10	11.6%	

In parallel, we evaluated MAT1A expression levels in a panel of NSCLC cell lines (A549, NCI-H1299, NCI-H1437, and NCI-H1944) and compared them to a lung epithelial cell line (BEAS-2B). Consistently, the NSCLC cell lines exhibited significantly elevated MAT1A expression compared to the non-malignant BEAS-2B cells (*p* < 0.05), as shown in Fig. 1E. Collectively, these data provide compelling evidence for the abnormal upregulation of MAT1A in NSCLC, implicating its involvement in the pathogenesis or progression of this malignancy.

### Correlation between MAT1A and clinicopathological features of NSCLC

Upon evaluating the IHC staining scores, MAT1A expression levels exceeding the median cutoff value of 6 were designated as high, while those below this threshold were considered low (Fig. 1D). To investigate the potential clinical significance of MAT1A expression in NSCLC, we conducted statistical analyses to ascertain its associations with clinicopathological features. Employing the Mann-Whitney U test, we identified significant associations between upregulated MAT1A expression and both tumor size (*p* = 0.041) and tumor stage (*p* = 0.013), as detailed in Table 2. Concordantly, these findings were further substantiated by Pearson correlation analysis, which confirmed a positive correlation between MAT1A expression and both tumor size (*p* = 0.040) and tumor stage (*p* = 0.012), as summarized in Supplementary Table S4.

**Table 2:** Relationship between MAT1A expression and tumor characteristics in patients with lung cancer.

Features	No. of patients	MAT1A expression		*p* value
		low	high	
All patients	77	34	43	
Age (years)				0.479
<62	35	17	18	
≥62	42	17	25	
Gender				0.674
Male	54	23	31	
Female	23	11	12	
Tumor size				0.041
<3.5 cm	33	19	14	
≥3.5 cm	44	15	29	
Stage				0.013
I	21	12	9	
II	25	14	11	
III	25	7	18	
IV	6	1	5	
T Infiltrate				0.196
T1	33	18	15	
T2	21	7	14	
T3	23	9	14	
Lymphatic metastasis (N)				0.252
N0	45	22	23	
N1	8	4	4	
N2	24	8	16	
Metastasis (M)				0.264
M0	72	33	39	
M1	5	1	4	

Furthermore, Kaplan-Meier survival analysis coupled with a log-rank test revealed that NSCLC patients exhibiting high MAT1A expression had significantly shorter survival durations compared to those with low MAT1A expression, indicative of a notable negative correlation between MAT1A expression and patient survival (*p* = 0.028) (Fig. 1F). Collectively, these results underscore the overexpression of MAT1A in NSCLC and its potential association with adverse prognostic outcomes in this patient population.

### MAT1A depletion inhibits malignant progression of NSCLC in vitro and in vivo

**Fig. 2:**
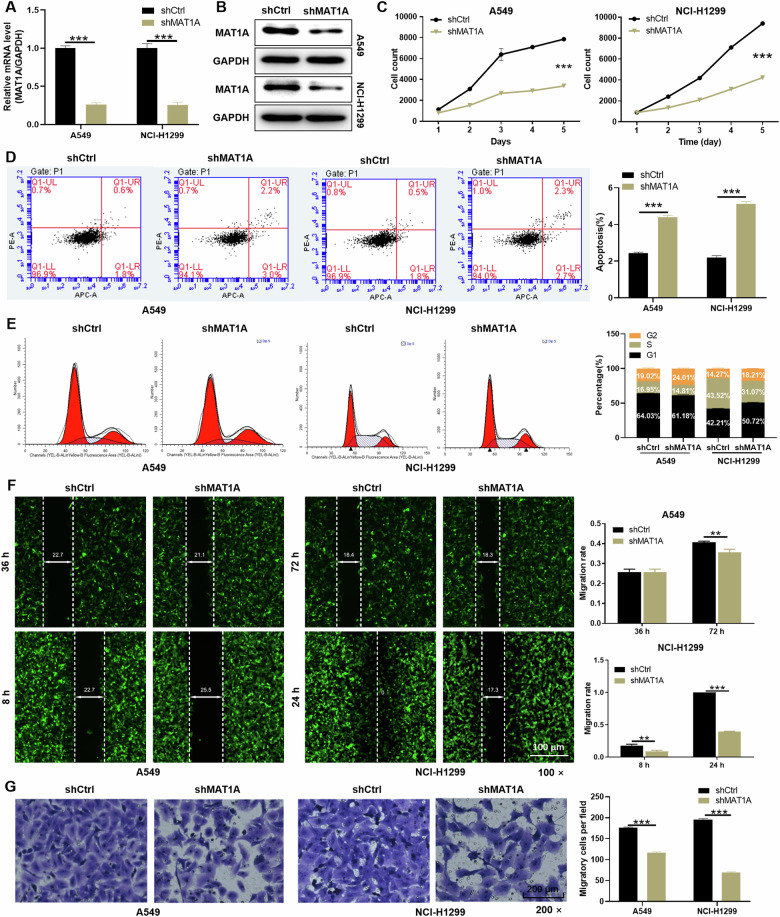
MAT1A depletion inhibited malignant phenotypes of NSCLC cells in vitro. **A** MAT1A mRNA levels and **B** protein levels in A549 and NCI-H1299 cells transfected with or without shMAT1A lentivirus were determined by qPCR and WB assays, respectively. GAPDH was used as inner control. **C** Cell viability of A549 and NCI-H1299 cell after MAT1A depletion was determined by Celigo cell count assays. **D** Cell apoptosis in A549 and NCI-H1299 cell after MAT1A depletion were analyzed by flow cytometry. **E** Cycle arrest in A549 and NCI-H1299 cell after MAT1A depletion were analyzed by flow cytometry. Cell migration in A549 and NCI-H1299 cell after MAT1A depletion was assessed by (**F**) wound-healing assay and (**G**) transwell assays. Results were shown as mean ± SD. **p < 0.01, ***p < 0.001.

To investigate the functional implications of MAT1A in NSCLC, we targeted two NSCLC cell lines, A549 and NCI-H1299, and specifically depleted MAT1A expression through transfection with custom-designed small interfering RNAs (shRNAs). Subsequent validation using qPCR and Western blot (WB) assays confirmed the successful downregulation of MAT1A at both the mRNA and protein levels in both A549 and NCI-H1299 cells (Fig. 2A, B).

Utilizing these MAT1A-knockdown cell models, we delved into the effects of MAT1A on the malignant phenotypes of NSCLC cells. In cell viability assays, we observed a marked reduction in the growth rate of both A549 and NCI-H1299 cells upon MAT1A depletion, compared to their respective negative control cells (*p* < 0.001) (Fig. 2C). Furthermore, analysis of apoptosis revealed a significant increase in the percentage of apoptotic cells in the shMAT1A group for both NSCLC cell lines, in stark contrast to the shCtrl group (*p* < 0.001) (Fig. 2D). Intriguingly, we also noted a pronounced accumulation of cells in the G2 phase of the cell cycle in both cell lines following infection with the shMAT1A lentivirus (*p* < 0.001), hinting at a cell cycle arrest induced by MAT1A depletion (Fig. 2E). To assess the impact on migratory abilities, we employed wound-healing and transwell migration assays. Our findings demonstrated that the knockdown of MAT1A significantly impaired the migratory capacities of NSCLC cells compared to their negative controls (*p* < 0.01), suggesting a crucial role for MAT1A in promoting the migratory potential of NSCLC cells (Fig. 2F, G).

To further elucidate the influence of MAT1A on tumor growth, we established xenograft tumor models in nude mice by subcutaneously injecting NCI-H1299 cells with MAT1A knockdown (shMAT1A) and negative control (shCtrl). After 79 days of monitoring, we observed a significant reduction in tumor growth in mice injected with MAT1A-knockdown cells compared to controls (*p* < 0.001) (Supplementary Fig. S1A). This finding was corroborated by fluorescence imaging and tumor body photographs on day 79, which also showed weaker tumor growth in the MAT1A-knockdown group (*p* < 0.05) (Supplementary Fig. S1B, C). Consistent with these results, Ki67, a key proliferative marker, was downregulated in the MAT1A-knockdown group, indicating inhibited proliferation (Supplementary Fig. S1D). In summary, these findings highlight the inhibitory effects of MAT1A knockdown on key malignant features of NSCLC cells, including proliferation, migration, and tumorigenic capacity.

### CCND1 as a key functional partner of MAT1A in driving NSCLC progression

**Fig. 3:**
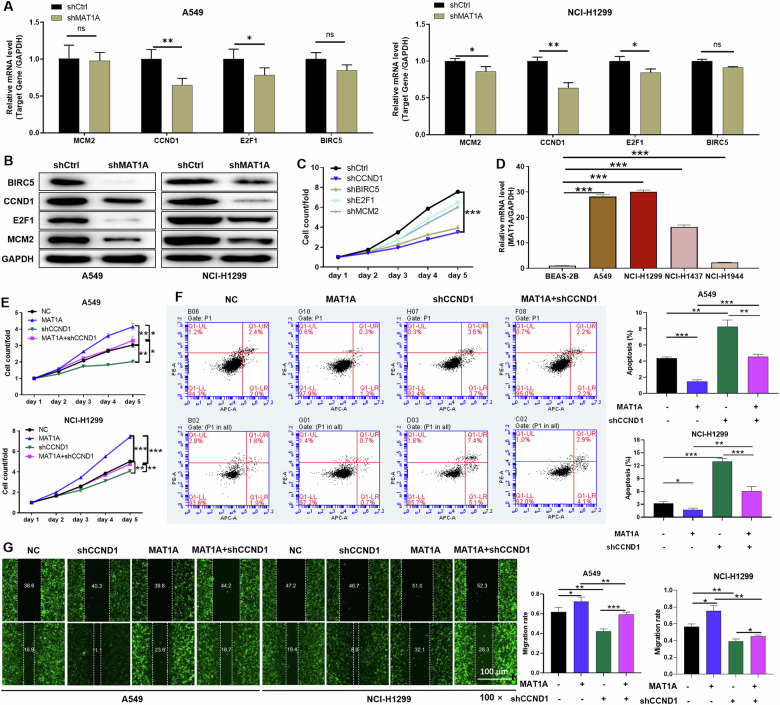
MAT1A facilitated NSCLC cell proliferation and migration through CCND1. **A** Relative mRNA levels of top 4 DEGs were detected by qPCR analysis in A549 and NCI-H1299 cell after MAT1A depletion. **B** Relative protein levels of CCND1, BIRC5, E2F1 and MCM2 in A549 and NCI-H1299 cell after MAT1A depletion were determined by WB assays. **C** Cell viabilities of A549 cell after depletion of CCND1, BIRC5, E2F1 and MCM2 were assessed by Celigo cell count assays at indicated times. **D** CCND1 expression levels in human NSCLC cell lines (A549, NCI-H1299 and NCI-H1944) and normal human lung epithelial cell line (BEAS-2B) were determined by qPCR. A549 and NCI-H1299 cells were grouped as NC (empty vector, as negative control), MAT1A (MAT1A-overexpressing), shCCND1 (CCND1 knockdown) and MAT1A+shCCND1 (MAT1A-overexpressing and CCND1-knockdown) groups, performed (**E**) Celigo cell count assays, (**F**) flow cytometry, (**G**) wound-healing assays. Results were shown as mean ± SD. *p < 0.05, **p < 0.05, ***p < 0.001.

To explore the functional partners of MAT1A, the RNA-seq was performed based on the MAT1A stable knockdown (*n* = 3) and control (*n* = 3) NCI-H1299 cell samples. Our results suggested that a total of 882 genes were upregulated and 1371 genes were downregulated genes along with MAT1A depletion (Supplementary Fig. S2A). By qPCR analysis, we validated the relative mRNA levels of top-ranked 4 differentially expressed genes (DEGs), and further confirmed the downregulation of BIRC5, CCND1, E2F1 and MCM2 at protein levels upon MAT1A depletion (Fig. 3A, B). Subsequently, the expression of these genes was knocked down under the mediation of shRNA (shBIRC5, shCCND1, shE2F1 and shMCM2), and performed proliferation detection. Compared with other targeted sequences, shCCND1 interference had the most significant inhibition on cell proliferation (*p* < 0.001) (Fig. 3B). Therefore, CCND1 was considered as a potential downstream target protein. Moreover, the expression level of CCND1 in NSCLC cell lines (A549, NCI-H1299, NCI-H1437 and NCI-H1944) was significantly higher than that in lung epithelial cell line (*p* < 0.001) (Fig. 3D).

To evaluate the regulatory role of MAT1A and CCND1 in NSCLC, overexpression of MAT1A and knockdown of CCND1 were constructed in A549 and NCI-H1299 cell lines, respectively, and functional recovery experiments were performed. MAT1A overexpression obviously enhanced cell viability of A549 and NCI-H1299 (*p* < 0.01), while which was attenuated when CCND1 was depleted (*p* < 0.05) (Fig. 3E). Moreover, the downregulation of CCND1 reversed the inhibitory effects of MAT1A on cell apoptosis of A549 and NCI-H1299 (*p* < 0.05) (Fig. 3F). Besides, the promotion of cell migration by MAT1A overexpression were also compromised due to knockdown of CCND1 (Fig. 3G). Collectively, these rescue experiments suggested that CCND1 serve as an essential partner of MAT1A contributing to NSCLC progression.

### MAT1A stabilizes CCND1 expression by competitively inhibiting SKP2 binding

**Fig. 4:**
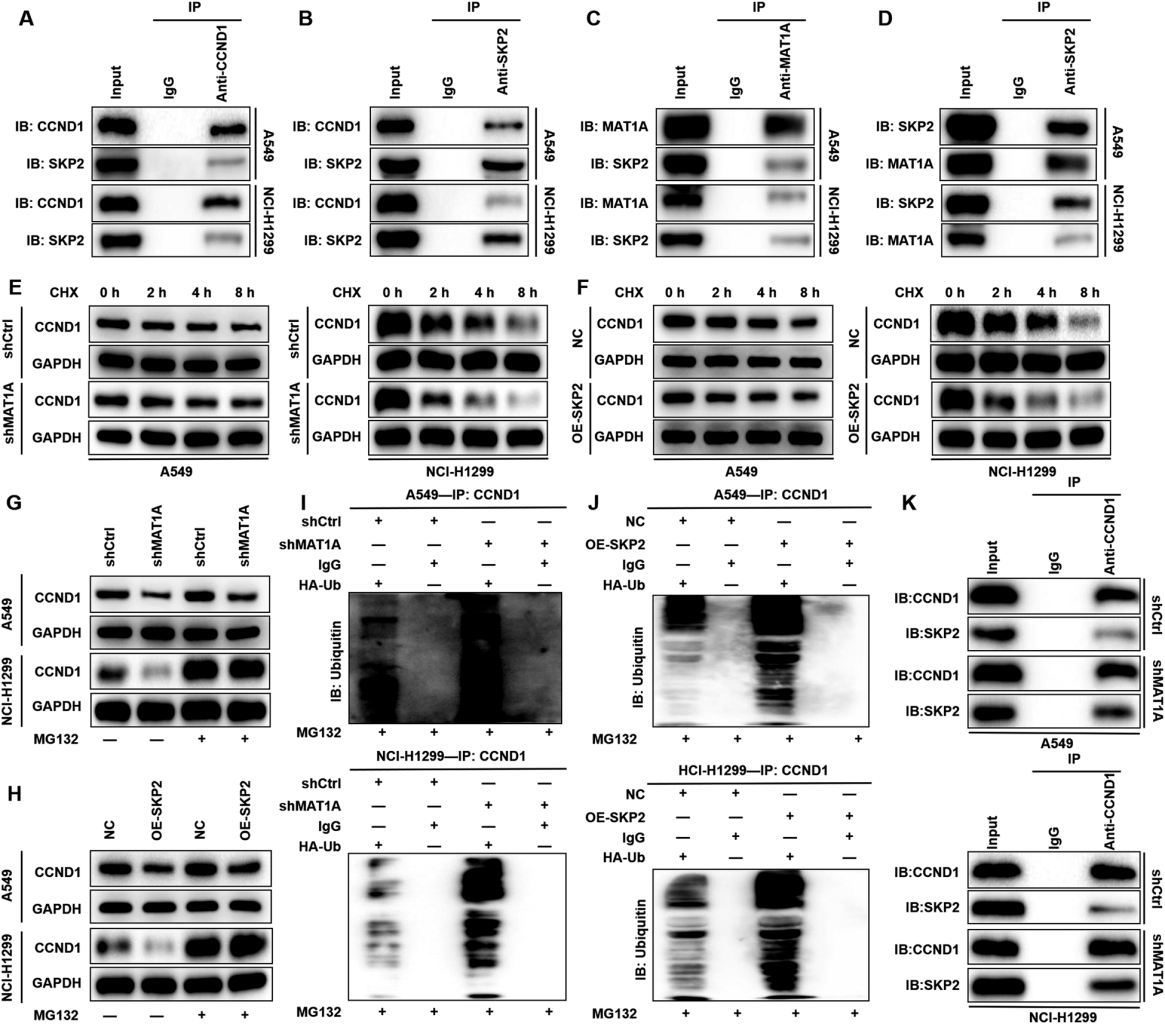
MAT1A inhibited the ubiquitination of CCND1 by blocking the binding of SKP2 to CCND1. In co-immunoprecipitation experiments, the total lysates of A549 and NCI-H1299 cells were immunized with (**A**) anti-CCND1 or (**B**) anti-SKP2 and WB, respectively. In the co-immunoprecipitation experiments, total lysates of A549 and NCI-H1299 cells were immunized with (**C**) anti-MAT1A or (**D**) anti-SKP2 and WB. **E** MAT1A knockdown and control A549 and NCI-H1299 cells were treated with protein synthesis inhibitor (CHX, cycloheximide from microbial, 10 μmol/L) and then subjected to cycloheximide chase assays. **F** SKP2 overexpressed and control A549 and NCI-H1299 cells were treated with CHX (10 μmol/L) to detect the relative protein levels of CCND1 at indicated time points. CCND1 protein level was determined in (**G**) MAT1A-depleted or (**H**) SKP2-overexpresed A549 and NCI-H1299 cells treated with or without proteasome inhibitor (MG132, 10 μmol/L). The ubiquitin of CCND1 immunoprecipitated in (**I**) MAT1A-depleted or (**J**) SKP2-overexpresed A549 and NCI-H1299 cells was detected. **K** Total lysates of A549 and NCI-H1299 cells with or without MAT1A knockdown were immunized with anti-CCND1 and WB.

As CCND1 is a crucial partner of MAT1A in driving NSCLC progression, elucidating their interactional mechanisms is imperative. Previous studies have linked MAT1A to the regulation of protein ubiquitination [18]. Furthermore, the Ubibrowser database predicts that CCND1 undergoes modification by the E3 ligase S-phase kinase-associated protein 2 (SKP2). Our CO-IP experiments demonstrated specific binding between CCND1 and SKP2, confirming their protein interaction (Fig. 4A, B). Notably, SKP2 also interacts with MAT1A (Fig. 4C, D), prompting the hypothesis that MAT1A regulates CCND1 ubiquitination via SKP2.

To test this, we silenced MAT1A in A549 and NCI-H1299 cells and treated them with a protein synthesis inhibitor (CHX). Subsequent cycloheximide chase assays revealed accelerated CCND1 degradation and reduced stability upon MAT1A knockdown (Fig. 4E). Similarly, SKP2 overexpression led to decreased CCND1 stability (Fig. 4F). Importantly, treatment with the proteasome inhibitor MG-132 partially rescued the destabilizing effects of MAT1A knockdown and SKP2 overexpression on CCND1 (Fig. 4G, H). These findings suggest that MAT1A modulates CCND1 stability through the ubiquitin-proteasome system (UPS), mediated by SKP2. Ubiquitination assays further confirmed that MAT1A knockdown and SKP2 overexpression significantly enhanced CCND1 ubiquitination, reinforcing our hypothesis that MAT1A regulates the ubiquitination of CCND1 under the mediation of SKP2 to stabilize its expression (Fig. 4I, J). Furthermore, MAT1A deficiency significantly enhanced the interaction between SKP2 and CCND1 (Fig. 4K), suggesting MAT1A controls CCND1 ubiquitination and degradation by modulating the SKP2-CCND1 complex. Taken together, these findings validate that MAT1A diminishes the ubiquitination degradation of CCND1 by competitively inhibiting the binding of SKP2 to CCND1, thereby enhancing the stability of CCND1 protein.

### The MAT1A/CCND1 signaling axis promotes glycolysis and tumorigenesis in NSCLC

The functional annotation of differentially expressed genes (DEGs) was conducted utilizing IPA, revealing a prominent association between these genes and metabolic pathways, particularly glycolysis, which was significantly repressed (Supplementary Fig. S2B). This observation prompted us to postulate a potential interplay between MAT1A and glycolysis in NSCLC.

**Fig. 5:**
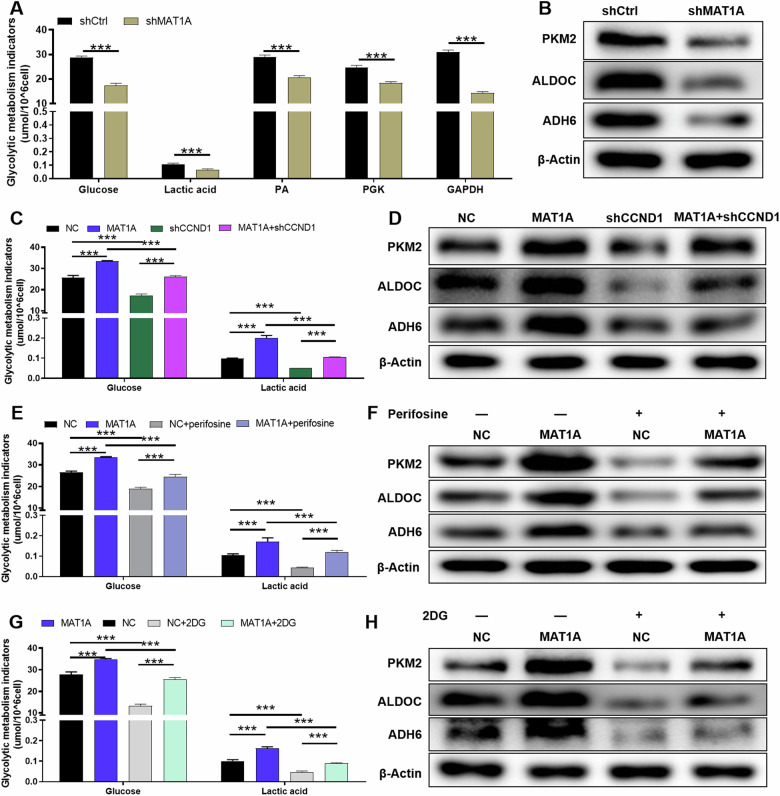
MAT1A enhances the glycolysis of NSCLC cells through CCND1. The metabolic profile of glycolysis in MAT1A knockdown A549 cells was analyzed, including (**A**) glucose utilization, lactic acid production, PA, PGK, GAPDH activity and (**B**) expression of pivotal glycolytic enzymes (PKM2, ALDOC, ADH6). **C**, **D** Measurement of glucose utilization, lactic acid production and (**D**) expression of pivotal glycolytic enzymes (PKM2, ALDOC, ADH6) in A549 cells (NC, as negative control; MAT1A-overexpressing; CCND1 knockdown; MAT1A-overexpressing and CCND1 knockdown). **E**, **F** MAT1A-overexpresed A549 cells treated with AKT inhibitor (perifosine) and measurement of (**E**) glucose utilization, lactic acid production and (**F**) expression of pivotal glycolytic enzymes (PKM2, ALDOC, ADH6). **G**, **H** MAT1A-overexpresed A549 cells treated with glycolysis inhibitor 2-deoxy-d-glucose (2-DG) and measurement of (**G**) glucose utilization, lactic acid production and (**H**) expression of pivotal glycolytic enzymes (PKM2, ALDOC, ADH6). Results were shown as mean ± SD. *p < 0.05, **p < 0.05, ***p < 0.001.

To substantiate this hypothesis, we assessed the comprehensive metabolic profile of glycolysis, including glucose utilization, lactic acid production, PA, PGK, GAPDH activity and expression of pivotal glycolytic enzymes (PKM2, ALDOC, ADH6). Our results demonstrated that MAT1A depletion in A549 cells significantly inhibited glycolysis (*p* < 0.001) (Fig. 5A, B). Intriguingly, CCND1 knockdown in A549 cells also impeded glycolysis compared to controls (*p* < 0.001), highlighting its involvement in glycolytic regulation (*p* < 0.001) (Fig. 5C, D). Rescue experiments corroborated this finding, revealing that CCND1 depletion partially reversed the glycolytic enhancement mediated by MAT1A overexpression (*p* < 0.001) (Fig. 5C, D).

Additionally, given the established role of the kinase AKT in tumor glycolysis, often overexpressed and facilitating glycolytic processes [19], we examined the effects of AKT inhibition (perifosine). This intervention potently suppressed the stimulatory effect of MAT1A overexpression on glycolysis in A549 cells (*p* < 0.001) (Fig. 5E, F). To further explore the regulatory mechanisms, we employed 2-deoxy-d-glucose (2-DG), a known inhibitor of glycolysis that disrupts d-glucose metabolism [20], and observed a marked attenuation of glycolysis induced by MAT1A overexpression following 2-DG treatment (*p* < 0.001) (Fig. 5G, H).

**Fig. 6:**
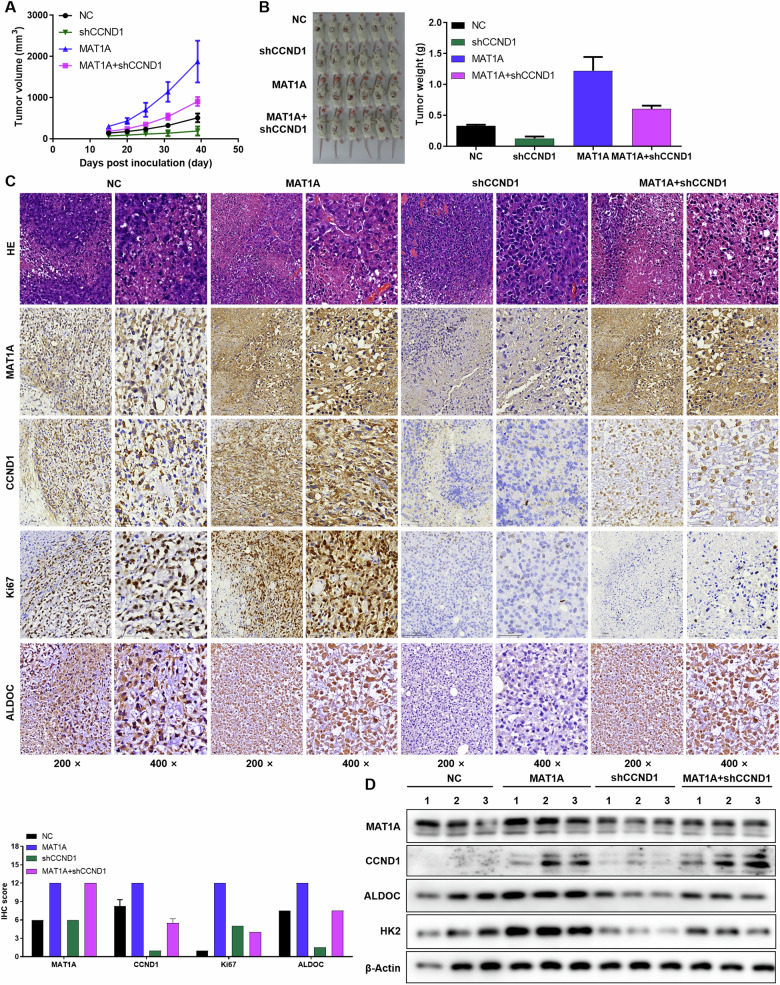
The MAT1A/CCND1 signaling axis promotes glycolysis and tumorigenesis in NSCLC. The xenograft tumor model in nude mice established by A549 cells that interfered with MAT1A and CCND1 expression. After 39 days of monitoring, **A** tumor volume and **B** tumor weight was measured. **C** HE, IHC staining and **D** WB analysis was performed to determine the expression of MAT1A, CCND1, proliferation marker Ki67 and glycolytic enzymes (ALDOC, HK2) after tumor tissue sections or protein extraction.

In addition, the xenograft tumor model in nude mice established by A549 cells that interfered with MAT1A and CCND1 expression reaffirmed the above results. Specifically, CCND1 knockdown partially reversed the stimulative effect of MAT1A overexpression on tumor growth in mice (Fig. 6A, B). HE, IHC staining and WB showed the expression of MAT1A, CCND1, proliferation marker Ki67 and glycolytic marker (ALDOC, HK2) in tumor tissues, emphasizing the regulation of MAT1A and CCND1 on NSCLC cells (Fig. 6C, D). Collectively, these findings underscore the essential partnership between MAT1A and CCND1 in orchestrating NSCLC glycolysis and tumorigenesis, providing novel insights into the molecular mechanisms underpinning this metabolic reprogramming in cancer.

## Discussion

In the realm of oncology research, the identification of biomarkers and therapeutic targets for NSCLC remains a pressing endeavor. Our study, which focused on elucidating the role and mechanisms of MAT1A in NSCLC, adds a significant piece to this complex puzzle. By leveraging the TCGA database and immunohistochemical staining, we have demonstrated a robust upregulation of MAT1A in NSCLC tissues, underscoring its potential as a prognostic indicator. The functional analyses conducted in our study, involving stable knockdown of MAT1A in NSCLC cell lines, revealed multifaceted effects on tumor behavior. The observed inhibition of proliferation, migration, and enhancement of apoptosis suggest that MAT1A plays a pivotal role in promoting the malignant phenotype of NSCLC cells. These findings align with previous studies that have implicated MAT1A in the regulation of cancer cell growth and survival pathways, albeit in different cancer types [10–14, 21]. However, our study provides a nuanced understanding of MAT1A’s specific mechanisms in NSCLC, which may inform the development of tailored therapeutic strategies.

Furthermore, Cyclin D1 (CCND1) was regarded as the downstream target of MAT1A to regulate NSCLC. As we all known, CCND1 was generally highly expressed in human tumors [22]. The abnormally high expression of CCND1 in malignant tumors could lead to chromosomal rearrangement and abnormal gene amplification, suggesting that CCND1 may play an important role in tumorigenesis [23–25]. Our data indicated that MAT1A overexpression could promote proliferation and migration of NSCLC cells and inhibited apoptosis, which could be partially alleviated by CCND1 knockdown. These results clearly revealed that CCND1 was a key downstream target for MAT1A regulation of cellular behaviors and further supported the tumor-promoting role of CCND1 in tumor progression.

As we all known, ubiquitination is involved in the regulation of various life activities such as cell cycle, proliferation, apoptosis, metastasis, gene expression, signal transmission, and damage repair [26]. Especially, ubiquitination is closely related to the pathogenesis of tumors and has become a new target for the development of new drugs [27]. In this study, we identified the E3 ubiquitin ligase S-phase kinase-associated protein 2 (SKP2, also known as FBXL1 or p45) was a potential substrate of CCND1. It was reported that SKP2 is a well‐characterized cancer‐related protein, whose stability was regulated by ubiquitin-proteasome system (UPS) during the cell cycle [28]. Of note, UPS is the main pathway of protein degradation in cells and participated in the degradation of more than 80% of proteins in cells [29]. Mechanistically, our discovery of the MAT1A-SKP2-CCND1 axis represents a novel insight into the regulation of cell progression in NSCLC. By accelerating CCND1 degradation through SKP2-mediated ubiquitination, MAT1A depletion disrupts the delicate balance between cell proliferation and apoptosis, thereby impairing tumor growth. In addition to SKP2, TRIM25, a member of the tripartite motif-containing (TRIM) family, increases nuclear factor erythroid 2-related factor (NRF2) through KEAP1 ubiquitination. NRF2 and KEAP1 alterations and mutations often occur in NSCLC. He et al., reported that NRF2 regulates the expression of genes encoding enzymes involved in various metabolic pathways [30]. Furthermore, Ding et al., found that the anti-oncogene of ring finger protein 180 (RNF180) downregulated C-myc through ubiquitin-dependent degradation inhibited the proliferation and glycolytic activity of NSCLC cells [31]. Tumor cells rely on highly active glycolysis and strong increase in lactic acid production to obtain a large amount of ATP to create a suitable microenvironment for the survival of tumor cells to meet the needs of rapid growth and proliferation of tumor cells [32]. In addition, tumor cells need to increase their efficiency by increasing glucose transporters, or various key enzymes, such as hexokinase 2 (HK2), pyruvate kinase M2 (PKM2), lactate dehydrogenase A (LDHA), aldolase C (ALDOC), alcohol dehydrogenase gene (ADH6) [32]. Peng et al. proposed that ubiquitin carboxyl-terminal hydrolase 47 (USP47) promotes glycolysis by stabilizing HK2 to induce the development of NSCLC [33]. Brand et al. found that production of associated lactic acid in response to LDHA impaired tumor immune surveillance by T cells and NK cells [34]. In this study, the identification of CCND1 as a critical MAT1A partner in orchestrating glycolytic metabolism highlights the importance of metabolic reprogramming in NSCLC progression. This finding resonates with the growing body of evidence that links metabolic alterations to cancer development and progression, emphasizing the need for a holistic approach in targeting cancer metabolism.

While our study sheds new light on the role of MAT1A in NSCLC, it is important to acknowledge its limitations. Firstly, the use of cell lines as model systems may not fully recapitulate the complexity of in vivo tumor microenvironments. Future studies utilizing patient-derived xenografts or organoids would provide a more physiologically relevant platform for validating our findings. Secondly, the molecular mechanisms underlying the MAT1A-SKP2-CCND1 axis and its impact on glycolytic metabolism require further interrogation, could help elucidate the upstream regulators and downstream effectors of this pathway.

In conclusion, our study establishes MAT1A as a promising prognostic marker and potential therapeutic target in NSCLC. The identification of the MAT1A-SKP2-CCND1 axis and its role in glycolytic metabolism provides a compelling rationale for targeting this pathway in the development of novel anticancer therapies.

## Supplementary information

Supplemental Figures and tables

Original data

## Data Availability

The data used and analyzed during the current study are available from the corresponding author on reasonable request.
