# Supplementary material for: MAT1A activation of glycolysis to promote NSCLC progression depends on stabilizing CCND1
Source: Cell Death Dis. 2024 Oct 22;15(10):768. doi: 10.1038/s41419-024-07113-7 (PMC11496809; doi:10.1038/s41419-024-07113-7)
Supplement: Supplementary file 1 — Supplemental Figures and tables [file 41419_2024_7113_MOESM1_ESM.docx]

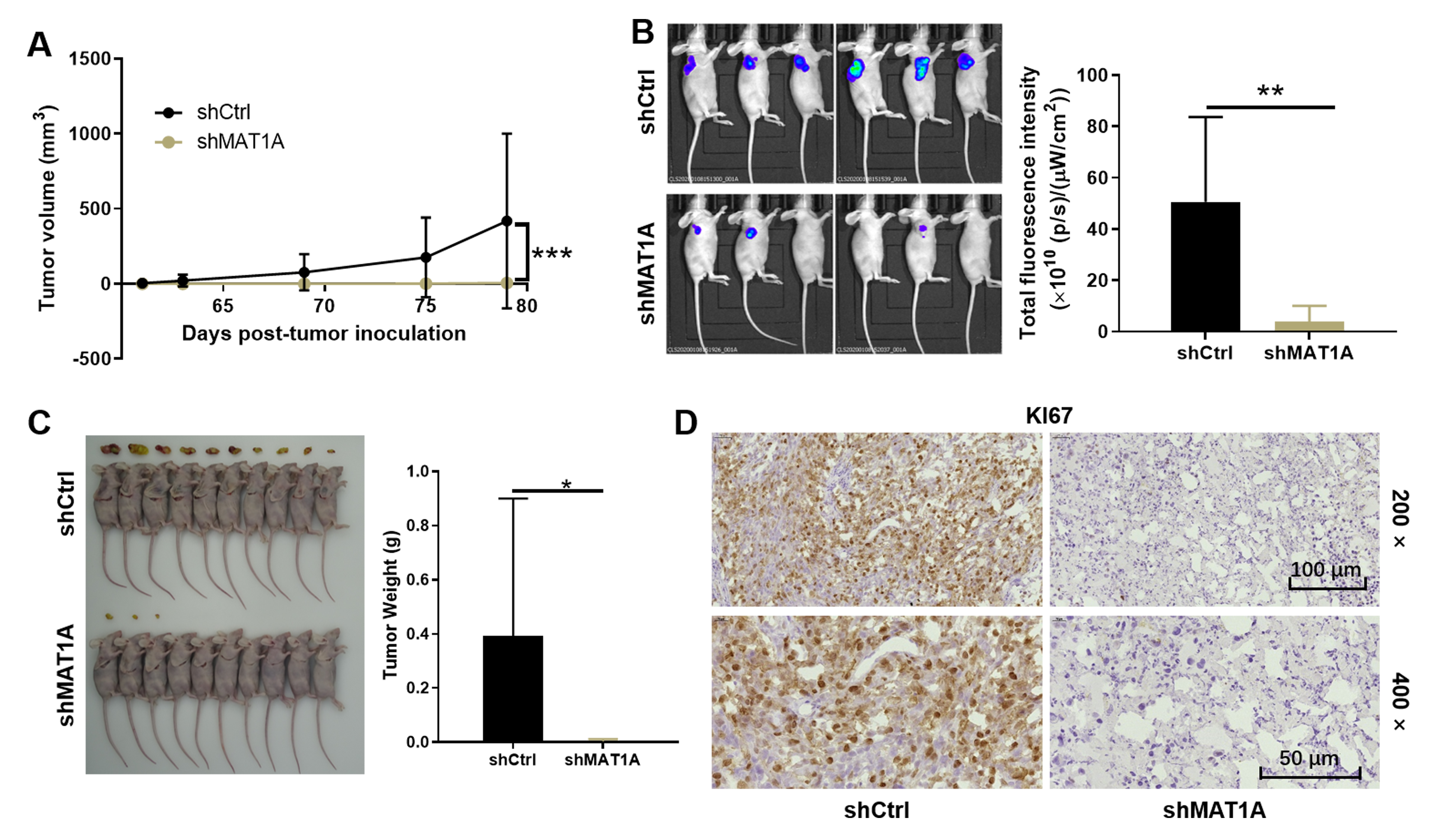


**Figure S1. MAT1A depletion inhibited** **the tumorigenic ability of NSCLC cells.**

(A-D) NCI-H1299 cells with MAT1A knockdown (shMAT1A) and negative control (shCtrl) were subcutaneously injected into nude mice to establish the xenograft tumor models. After 79 days of monitoring, (A) the tumor volume, (B) fluorescence intensity and (C) weight were measured. Results were shown as mean ± SD. ^**^p <0.01, ^***^p<0.001. (D) Tumor tissue was sliced for IHC staining to detect the expression of proliferation marker Ki67.


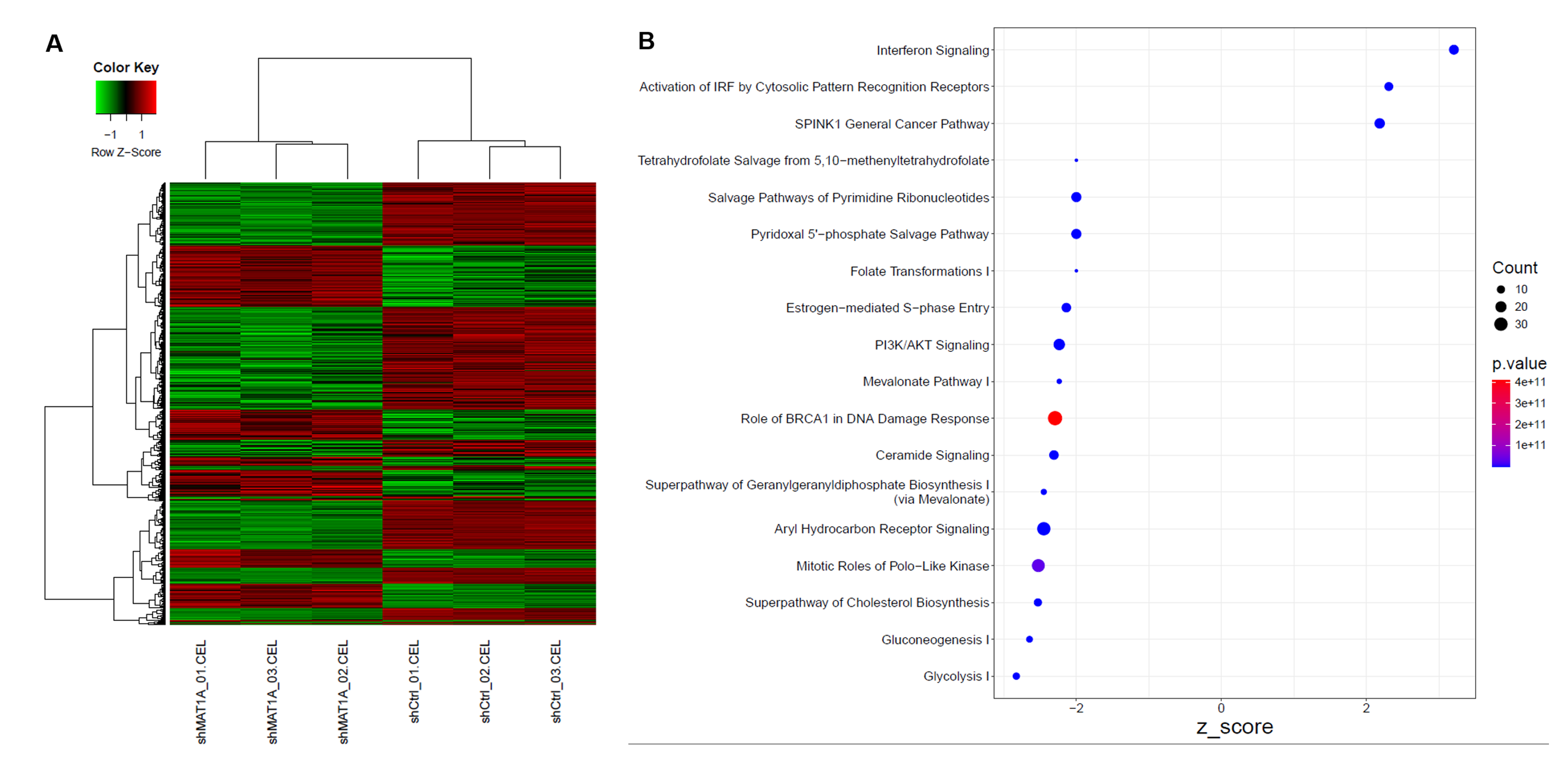


**Figure S2. RNA sequencing and pathway enrichment analysis of NSCLC cells.** (A)The Hierarchical Clustering showed the expression profiles of differentially expressed genes (DEGs) between shCtrl (n=3) and shMAT1A (n=3) NCI-H1299 cells (|Fold Change|≥1.3 and p<0.05). Red indicates upregulated genes, green indicates downregulated genes. (B) The functional enrichment of DEGs was annotated using IPA analysis. Red indicates promotion; Blue indicates inhibition.

**Table S1** **The shRNA target sequences.**

| Gene | Number | Target shRNA sequences (5'-3') |
| --- | --- | --- |
| MAT1A | MAT1A-1 | TTGCAACGTGCTGGTGGCTTT |
| MAT1A | MAT1A-2 | GGGAGAGGGACACCCGGATAA |
| MAT1A | MAT1A-3 | CACCTCAATGGCCATGGTGGA |
| CCND1 | CCND1-1 | GGTGAACAAGCTCAAGTGGAA |
| CCND1 | CCND1-2 | CCACAGATGTGAAGTTCATTT |
| CCND1 | CCND1-3 | GGTGAACAAGCTCAAGTGGAA |

**Table S2** **Primer sequences in** **qPCR.**

| Primer name | Forward primer sequence (5’-3’) | Reverse primer sequence (5’-3’) |
| --- | --- | --- |
| GAPDH | TGACTTCAACAGCGACACCCA | CACCCTGTTGCTGTAGCCAAA |
| MAT1A | ATGCCCCTCACCATCATCCT | ATGACTGCGCCATTGTCCTG |
| E2F1 | CACTTTCGGCCCTTTTGCTC | GTGCTCTCACCGTCCTACAC |
| BIRC5 | TCTCAAGGACCACCGCATCT | TTTGCATGGGGTCGTCATCT |
| CCND1 | AGCTGTGCATCTACACCGAC | GAAATCGTGCGGGGTCATTG |
| MCM2 | CCAATGGCTTCCCTGTCTT | TCATCGGTCAGTTCCCCTAC |

**Table S3 Antibodies applied in Western blot, Co-IP, and Ubiquitination assays.**

| Assay | Primary antibody | Size/kDa | Diluted multiples | Source | Company | Catalog No. |
| --- | --- | --- | --- | --- | --- | --- |
| WB | MAT1A | 44 | 1:1000 | Rabbit | Abcam | ab129176 |
|  | BIRC5 | 16 | 1:1000 | Rabbit | Abcam | ab469 |
|  | CCND1 | 36 | 1:2000 | Rabbit | CST | 2978 |
|  | E2F1 | 47 | 1:2000 | Rabbit | Proteintech | 12171-1-AP |
|  | MCM2 | 102 | 1:2000 | Rabbit | Proteintech | 10513-1-AP |
|  | GAPDH | 36 | 1:30000 | Mouse | Proteintech | 60004-1-lg |
| Co-IP | CCND1 | 34 | 1:2000 | Rabbit | Abcam | ab134175 |
|  | SKP2 | 48 | 1:1000 | Rabbit | CST | 2652S |
|  | MAT1A | 44 | 1:1000 | Rabbit | Abcam | ab129176 |
|  | Ubiquitin |  | 1:1000 | Mouse | Santa Cruz | sc-47721 |
|  | Secondary antibody |  | Diluted multiples |  | Company | Catalog No. |
|  | Goat Anti-Rabbit |  | 1:3000 |  | Beyotime | A0208 |
|  | Goat Anti-Mouse |  | 1:3000 |  | Beyotime | A0216 |

**Table S4. Spearman correlation analysis between MAT1A expression and clinicopathologic characteristics in patients with lung cancer.**

|  |  | MAT1A |
| --- | --- | --- |
| Tumor size | Spearman correlation | 0.234 |
|  | Significance (two-tailed) | 0.040 |
|  | N | 77 |
| Stage | Spearman correlation | 0.284 |
|  | Significance (two-tailed) | 0.012 |
|  | N | 77 |
